# Phytosterols and Cardiovascular Risk Evaluated against the Background of Phytosterolemia Cases—A German Expert Panel Statement †

**DOI:** 10.3390/nu15040828

**Published:** 2023-02-06

**Authors:** Eberhard Windler, Frank-Ulrich Beil, Heiner K. Berthold, Ioanna Gouni-Berthold, Ursula Kassner, Gerald Klose, Stefan Lorkowski, Winfried März, Klaus G. Parhofer, Jogchum Plat, Günter Silbernagel, Elisabeth Steinhagen-Thiessen, Oliver Weingärtner, Birgit-Christiane Zyriax, Dieter Lütjohann

**Affiliations:** 1 Preventive Medicine, University Heart Center, University Hospital Hamburg-Eppendorf, Hamburg-Eppendorf, Martinistr. 52-Bldg. N26, 20246 Hamburg, Germany; 2 Ambulanzzentrum, Universitätsklinikum Hamburg-Eppendorf, Martinistr. 52, 20246 Hamburg, Germany; 3 Department of Internal Medicine and Geriatrics, Bethel Clinic, 33611 Bielefeld, Germany; 4 Center for Endocrinology, Diabetes and Preventive Medicine, Faculty of Medicine and University Hospital Cologne, University of Cologne, Kerpener Str. 62, 50937 Cologne, Germany; 5 Lipid Clinic at the Interdisciplinary Metabolism Center, Charite-Universitätsmedizin Berlin, Augustenburger Platz 1, 13353 Berlin, Germany; 6 Praxen Dres. T. Beckenbauer & S. Maierhof, Am Markt 11, 28195 Bremen und Dres. I. van de Loo & K. Spieker, Gerold Janssen Straße 2 A, 28359 Bremen, Germany; 7 Institute of Nutritional Science and Competence Cluster for Nutrition and Cardiovascular Health (nutriCARD), Halle-Jena-Leipzig, Friedrich Schiller University Jena, Dornburger Str. 25, 07743 Jena, Germany; 8 SYNLAB Akademie für Ärztliche Fortbildung, SYNLAB Holding Deutschland GmbH, P5,7, 68161 Mannheim, Germany; 9 Medical Clinic V, Medical Faculty Mannheim, University of Heidelberg, Theodor-Kutzer-Ufer 1-3, 68167 Mannheim, Germany; 10 Clinical Institute of Medical and Chemical Laboratory Diagnostics, Medical University of Graz, 8010 Graz, Austria; 11 Medizinische Klinik IV, Klinikum der Universität München, Grosshadern, Marchioninistr. 15, 81377 München, Germany; 12 Department of Nutrition and Movement Sciences, School of Nutrition and Translational Research in Metabolism (NUTRIM), Maastricht University, 6211 LK Maastricht, The Netherlands; 13 Division of Vascular Medicine, Department of Internal Medicine, Medical University of Graz, 8010 Graz, Austria; 14 Arbeitsbereich Lipidstoffwechsel der Medizinischen Klinik für Endokrinologie und Stoffwechselmedizin, Charité—Universitätsmedizin Berlin, Augustenburger Platz 1, 13353 Berlin, Germany; 15 Klinik für Innere Medizin I, Universitätskliniken Jena, Friedrich-Schiller-Universität Jena, 07743 Jena, Germany; 16 Midwifery Science—Health Care Research and Prevention, Research Group, Preventive Medicine and Nutrition, Institute for Health Services Research in Dermatology and Nursing (IVDP), University Medical Center Hamburg-Eppendorf, Martinistr. 52, 20246 Hamburg, Germany; 17 Institute of Clinical Chemistry and Clinical Pharmacology, University Clinics Bonn, 53127 Bonn, Germany

**Keywords:** plant sterols, phytosterols, phytosterolemia, atherosclerosis, cardiovascular disease, cholesterol absorption

## Abstract

Phytosterols (PSs) have been proposed as dietary means to lower plasma LDL-C. However, concerns are raised that PSs may exert atherogenic effects, which would offset this benefit. Phytosterolemia was thought to mimic increased plasma PSs observed after the consumption of PS-enriched foods. This expert statement examines the possibility of specific atherogenicity of PSs based on sterol metabolism, experimental, animal, and human data. Observational studies show no evidence that plasma PS concentrations would be associated with an increased risk of atherosclerosis or cardiovascular (CV) events. Since variants of the ABCG5/8 transporter affect the absorption of cholesterol and non-cholesterol sterols, Mendelian randomization studies examining the effects of ABCG5/8 polymorphisms cannot support or refute the potential atherogenic effects of PSs due to pleiotropy. In homozygous patients with phytosterolemia, total PS concentrations are ~4000% higher than under physiological conditions. The prevalence of atherosclerosis in these individuals is variable and may mainly relate to concomitant elevated LDL-C. Consuming PS-enriched foods increases PS concentrations by ~35%. Hence, PSs, on a molar basis, would need to have 20–40 times higher atherogenicity than cholesterol to offset their cholesterol reduction benefit. Based on their LDL-C lowering and absence of adverse safety signals, PSs offer a dietary approach to cholesterol management. However, their clinical benefits have not been established in long-term CV endpoint studies.

## 1. Introduction

Phytosterolemia, formerly also known as sitosterolemia, is a rare, autosomal-recessively transmitted inborn disorder of metabolism caused by mutations of the genes encoding the adenosine triphosphate (ATP)-binding cassette (ABC) G5/8 co-transporter [1,2]. The disease is often advocated to illustrate the possible consequences of an increased plasma concentration of phytosterols (PSs), also known as plant sterols and stanols, due to an increased dietary intake from foods with added PSs. Typically, daily PS intake is about 300 mg with a habitual diet and can be as high as 600 mg/d in vegetarians [3]. The recommended intake of PSs to lower LDL-C is around 2 g/d. The two most common PSs are sitosterol and campesterol.

Selective observations of individual cases with severe atherosclerosis, especially in children and adolescents affected by phytosterolemia, have provided reasonable grounds to suspect that PSs may advance atherosclerosis as much as or even more severely than LDL-cholesterol (LDL-C), a causal risk factor for atherosclerosis and cardiovascular disease (CVD) [4]. While PSs reduce LDL-C concentrations [3], concerns remain that their atherogenic effects may outweigh their benefits.

In fact, homozygous defects in the genes encoding the ABCG5/G8 tandem transporter, which causes phytosterolemia, modulate the absorption of about forty sterols, including cholesterol and non-cholesterol sterols (also known as xenosterols) with various complex effects on sterol and lipid metabolism and other tissue and organ functions. All these effects are unlikely to be associated with only a single sterol [5]. Furthermore, since the phenotype of phytosterolemia is highly variable, modifying influences must be considered.

This expert statement discusses the hypothesis of a specific atherogenicity of PSs based on the analysis of the current understanding of sterol metabolism, experimental and animal studies, epidemiological data from observational studies, and clinical experience in patients with phytosterolemia.

## 2. Regulation of Intestinal Absorption and Biliary Excretion of Phytosterols

The absorption and excretion of PSs, as well as of cholesterol (both from dietary and biliary sources), involves several membrane transporters and intracellular proteins. In principle, the organism is protected from absorption and accumulation of xenosterols such as PSs by redundant mechanisms [6]. Plasma concentrations of total PSs (mainly the sum of sitosterol and campesterol) are generally between 7–24 µmol/L (0.3–1.0 mg/dL), which is approximately 500-fold lower than circulating concentrations of total cholesterol; subsequently, PSs account for less than 1% of total plasma sterols [3].

The reason for the considerable difference between the plasma concentrations of cholesterol and PSs is twofold. First, PSs—in contrast to cholesterol—cannot be synthesized by mammalian cells, and second the selectivity of at least five successive absorption and excretion processes mediated via complex and not yet fully understood mechanisms prefer PSs over cholesterol [7]. As an example, the Niemann-Pick C1-like1 (NPC1L1) protein located on the membrane of the enterocytes binds cholesterol with a higher affinity than PSs [8,9,10]. PSs that are absorbed despite this first selection process are inhibited from incorporation into chylomicrons due to the specificity of two subsequent processes. Acyl-CoA cholesterol acyl transferase 2 (ACAT2) prefers cholesterol over PSs so that cholesteryl esters are predominantly formed and subsequently preferentially incorporated into chylomicrons [11,12]. Thus, the majority of PS remains unesterified for transfer back from the enterocytes into the intestinal lumen by the ABCG5/G8 transporter, which prefers PS over cholesterol [13,14]. This results in a total absorption rate of PSs of less than 5% [14]. In contrast, the average cholesterol absorption is about 54%, with a range from 20 to 80% [15,16].

**Figure 1:**
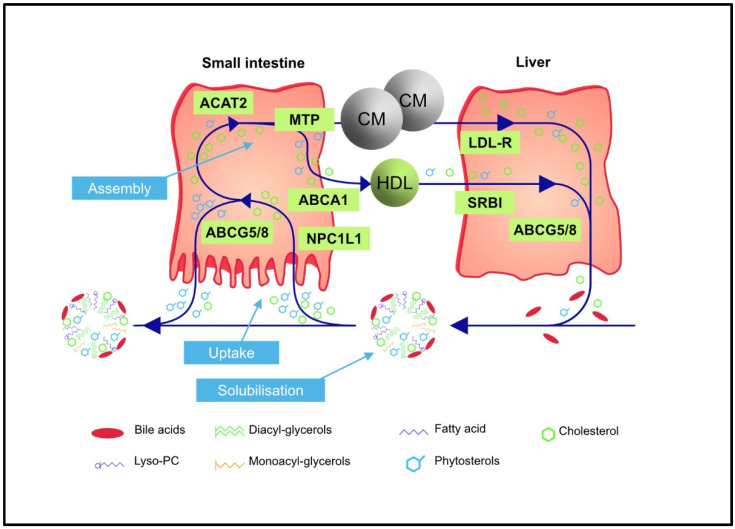
Flux and homeostasis of sterols (cholesterol and non-cholesterol sterols) with a focus on intestinal absorption and biliary secretion (adopted and modified from [26]. ABCA1, ATP binding cassette transporter A1; ABCG5/8, ATP binding cassette co-transporters G5 and G8; ACAT2, acetylcoenzyme-A cholesterol acyl transferase 2; CM, chylomicron; HDL, high-density lipoprotein; LDL-R, low-density lipoprotein receptor; MTP, microsomal triglyceride transfer protein; NPC1L1, Niemann Pick C1-like 1 protein; SRBI, scavenger receptor class B type 1.

The PSs remaining in the enterocytes are esterified with fatty acids in the same way as cholesterol by ACAT2 and then incorporated into chylomicrons. Next, PSs in chylomicrons and their remnants or in HDL particles are predominantly taken up into the liver. Here, PSs once again encounter the ABCG5/G8 transporter, which transfers them into the bile ducts for secretion with the bile. Some of the secreted PSs can be reabsorbed back into the liver through the NPC1L1 protein of the bile ducts. However, as in the intestine, there is a higher affinity for cholesterol than PS reabsorption [17]. These redundant protective selection mechanisms are so efficient that even heterozygous mutations in one of the heterodimers of the ABCG5/G8 transporter will result in only a modest increase in plasma PS concentrations of around 0.034 mmol/L (approx. 1.4 mg/dL) [18,19]. Even in the case of homozygous or combined heterozygous mutations of the ABCG5/G8 transporter, such as in phytosterolemia, PS plasma concentrations remain at about 0.3–1.2 mmol/L (approx. 12–48 mg/dL) [20,21]. Hence, PS concentrations never reach the level of plasma cholesterol concentrations despite a comparable dietary intake. The main steps involved in the intestinal absorption and biliary secretion of sterols (cholesterol and PSs) are depicted in Figure 1.

PSs are found in free and, more so, esterified forms in all serum lipoprotein fractions and in the bile [22,23,24]. Approximately two-thirds of plasma PSs are found in LDL particles, while HDL seems to make the largest contribution to the transport of PSs from the intestine to the liver [25].

## 3. Phytosterolemia—An Inherited Disorder of Sterol Transport

Clarification of the pathophysiology of phytosterolemia has improved our understanding of phytosterol metabolism [2]. Phytosterolemia is caused by homozygous or compound heterozygous defects in the genes ABCG5/sterolin-1 and ABCG8/sterolin-2 on chromosome 2p21 [27,28,29,30,31,32]. These genes encode the obligate heterodimeric ABC transporters, ABCG5 and ABCG8, with the result that a defect in one of the two monomers may cause a malfunction of the entire heterodimer. The ABCG5/G8 transporter pumps sterols, such as cholesterol, but preferably PSs, from the apical surface of the enterocytes back into the small intestine, as well as cholesterol and PSs from hepatocytes into the bile ducts [5,33]. The excretion of PSs from the hepatocytes into the bile canaliculi is necessary since, in mammalians, most PSs cannot be converted into bile acids and are subsequently excreted [34,35].

Phytosterolemia has been considered a rare inherited disease. Worldwide, only some 100 phytosterolemia cases have been described limiting possible correlations to clinical manifestations. However, based on recent advances in genetics, the prevalence of individuals with detrimental mutations in ABCG5/G8 genes may be more than 1 in 200,000 individuals [1]. Phytosterolemia may also be underdiagnosed because clinically it is difficult to distinguish clinically from familial hypercholesterolemia (FH).

### 3.1. Plasma Sterol Concentrations and Cardiovascular Disease in Phytosterolemia

Characteristic symptoms of phytosterolemia, including severe hypercholesterolemia, xanthomas, aortic stenosis, as well as fatal and non-fatal cardiac events, have been reported predominantly in infants/children up to the age of adolescence, whereas these complications are apparently not as frequent with increasing age. In adults, mostly low to average plasma cholesterol concentrations have been reported, but LDL-C levels similar to those in heterozygous FH have also been observed in some cases [36]. The pronounced cardiovascular (CV) manifestations of homozygous phytosterolemia in children and adolescents are possibly the result of extremely high plasma cholesterol concentrations of up to more than 20 mmol/L (770 mg/dL). It has been suggested that the very high cholesterol concentrations in childhood are caused by the immaturity of the mechanisms that limit sterol intake [37]. Apparently, the effect is accentuated by a higher cholesterol burden in breast-fed infants and high consumption of dairy products in childhood [38]. High LDL-C during childhood has been described in several case reports as reviewed previously [14]. For example, in an 11-year-old with phytosterolemia, the plasma sitosterol concentration was 0.7 mmol/L (approx. 28 mg/dL), but the tendinous and tuberous xanthomas were similar to homozygous FH. The total plasma sterol concentration was 555 mg/dL and was reduced to 221 mg/dL with a cholesterol-lowering diet [39]. Treatment with cholestyramine resulted in total sterols of 173 mg/dL, the tuberous xanthomas disappeared, and the tendinous xanthomas decreased, while plasma PSs remained elevated. A similar experience was reported in two Japanese sisters with phytosterolemia whose xanthomas disappeared after a cholesterol-lowering diet alone [40]. These cases underpin that cholesterol concentrations can be extremely high in childhood phytosterolemia and are most likely responsible for the atherosclerosis and CVD encountered in these patients [29,41].

The hypothesis of temporarily high blood cholesterol levels in phytosterolemia is supported by the observation of several adult patients with phytosterolemia without symptoms of atherosclerotic CVD whose plasma concentrations of cholesterol could be well controlled, while their plasma PS concentrations were reduced by ezetimibe but not normalized [42]. In a German outpatient lipid clinic, two clinically inconspicuous brothers with phytosterolemia were monitored until their ninth decade, whereas another brother died at the age of 20 of suspected FH [*personal communication Frank-Ulrich Beil*].

After the first description in 1974 [22], more and more reports have been published on patients with xanthomas and macrothrombocytopenia, homozygous or compound heterozygous for mutations in the ABCG5 or ABCG8 gene, and thus biochemically and genetically diagnosed phytosterolemia, but without evidence of clinically relevant atherosclerosis [33,43,44,45,46,47,48,49,50,51,52,53,54]. Even in a family of a 13-year-old boy with phytosterolemia and severe atherosclerotic disease who died from sudden cardiac death, some, but not all, affected relatives developed premature ischemic heart disease [55]. In two sisters and three unrelated patients with suspected homozygous FH at the ages of 4–8 years, phytosterolemia was diagnosed. Plasma concentrations of total cholesterol were between 409 and 805 mg/dL (10.6 and 20.8 mmol/L), and LDL-C was between 372 and 630 mg/dL (9.6 and 16.3 mmol/L) [56]. All these patients had xanthomas, and some had thrombocytopenia. While changes in the diet and treatment with cholestyramine and ezetimibe resulted in LDL-C concentrations of 58 and 96 mg/dL [1.5 and 2.5 mmol/L), sitosterol and campesterol remained approximately 10 times higher than normal, ranging between 12 and 29 mg/dL (0.48 and 1.9 mmol/L). However, up to the ages of 17–33 years, no clinical signs of atherosclerosis were found by ultrasound examinations of the carotids, by coronary angiography or by cardiac computed tomography (CT) calcium score. The carotid intima-media thickness (IMT) of the carotids remained approximately constant at 0.45–0.54 mm [56]. This may suggest that any damaging potential of plant sterols may be harnessed once LDL-C cholesterol is sufficiently controlled by drug treatment, but obviously, systematic and challengeable evidence for this is lacking.

A case of a 33-year-old female with chronic tendosynovitis, xanthoma, and elevated LDL-C had campesterol and sitosterol concentrations of 7–12-fold and 20–38-fold higher, respectively, above the upper limit of normal but showed no clinical evidence of premature atherosclerosis [54]. The absence of atherosclerosis may again suggest that phytosterolemia is not necessarily accompanied by premature vascular disease.

The mechanism underlying the high LDL-C concentrations in phytosterolemia has not been understood. Possibly, the inability to limit cholesterol absorption due to defective re-secretion of absorbed cholesterol into the gut lumen may increase the flux of intestinal cholesterol into the liver, which may, in turn, down-regulate hepatic LDL receptor expression. Typical for phytosterolemia, plasma cholesterol concentrations decrease drastically with a cholesterol-lowering diet. This was regarded as a diagnostic sign at the time before PSs were measured, and phytosterolemia was named pseudo-homozygous FH [40,55,57]. A Korean infant, exclusively breast-fed, presented with phytosterolemia and developed extensive intertriginous xanthomas in the third month of life. The infant’s serum total cholesterol (675 mg/dL or 17.5 mmol/L) and LDL-C (540 mg/dL or 14 mmol/L) decreased dramatically following a cholesterol-lowering diet and cholestyramine treatment. At three years of age, LDL-C was 118 mg/dL (3.1 mmol/L), but the plasma sitosterol concentration remained increased at 19 mg/dL (0.76 mmol/L, while the xanthomas had completely disappeared [58]. The infant’s five-year-old sister with the same PS phenotype was asymptomatic, suggesting that mechanisms other than the PS concentration should be responsible for the clinical manifestation of the disease.

The observed extreme plasma cholesterol elevations seen in childhood may not be encountered when the initial diagnosis is made later in life. It is conceivable that the development of atherosclerosis is initiated during childhood, resulting in CV events in childhood and young adulthood, at a time at which plasma PSs and cholesterol concentrations have already declined so that a definite correlation between events and plasma sterol concentrations is not apparent [42]. At an adult age, the regulatory mechanisms of sterol intake will become effective, resulting in a decrease of both PS and cholesterol intake and enabling the normalization of the plasma cholesterol concentration through dietary changes and targeted therapy. For example, a normocholesterolemic phytosterolemia patient with a total cholesterol concentration of 192 mg/dL (5.0 mmol/L) had to undergo bypass surgery at the age of 29. He was primarily diagnosed with FH, suggesting that he had very high LDL-C concentrations in his younger years and that during that time, major vascular damage had occurred [45].

Clinically, infants heterozygous for phytosterolemia are not affected, while homozygous infants may develop xanthomas of the patella and Achilles tendons, the extensor tendons of the hand, and tubero-eruptive xanthomas at the elbows, or other pressure-bearing areas. These xanthomas, however, may disappear with increasing age in response to diet and, if necessary, medical therapy [6,48,59]. Next, fatal and non-fatal coronary events, as well as aortic stenoses, have been reported, mainly in children from the age of five up to young adulthood [14,22,55,60].

### 3.2. Heterozygous Phytosterolemia and Plasma Phytosterol Concentrations

Due to the redundant defense mechanisms outlined above, heterozygotes for mutations in the genes of the ABCG5/G8 transporter are clinically asymptomatic, despite slightly elevated plasma PSs [6]. Whether plasma PS concentrations increase in affected individuals, who consume PS-enriched foods with their diet, has been repeatedly examined. For example, two studies with obligate heterozygotes for phytosterolemia consuming a low-fat diet (American Heart Association Step 1 Diet) containing 2.2 g/day of PSs reported an increase in average plasma PS concentrations from 0.9 mg/dL to 1.7 mg/dL (0.04 to 0.07 mmol/L), whereas LDL-C decreased by about 16 mg/dL (0.41 mmol/L) [61]. The individually highest levels for campesterol plus sitosterol of about 2 mg/dL (0.08 mmol/L) increased after intake of PS-enriched margarine to about 3.4 mg/dL (0.14 mmol/L). On average, campesterol concentrations roughly doubled, while sitosterol concentrations increased by about 50%. Similar results were also found in other studies with individuals heterozygous for phytosterolemia [62,63]. These changes are in the range of those reported for individuals without evidence of heterozygous phytosterolemia (an increase in sitosterol of 17–50% and in campesterol of 72–114%) [61].

At the same time, the effect of dietary PS intake on plasma LDL-C lowering in heterozygotes for phytosterolemia is comparable to that of non-genetically affected individuals and can even be significantly higher in the presence of certain mutations in the ABCG5/G8 and NPC1L1 genes [63]. For instance, in the ABCG8 1289 C > A (T400 K) polymorphism, the A allele carriers with high basal plasma PS concentrations demonstrated a 3.9-fold greater reduction in LDL-C than their low basal plasma PS counterparts [63]. It can therefore be speculated that individuals with increased intestinal sterol absorption due to heterozygous mutations in the ABCG5/G8 genes may benefit from a higher dietary PS intake at the extent of any unaffected individual (or even more) due to the reduction of cholesterol absorption. This is supported by the finding that individuals with high cholesterol absorption may benefit more from the inhibition of cholesterol absorption by PS supplementation [18,64,65].

In a recent study, rare heterozygous loss-of-function (LoF) variants in ABCG5 or ABCG8 were associated with blood lipids and CVD risk [66]. Compared to non-carriers, carriers of heterozygous LoF variants in ABCG5 had higher plasma sitosterol and ~25 mg/dL higher LDL-C and were at a two-fold higher risk of CAD [66]. Moreover, the impact of heterozygous LoF carrier status on CVD risk was proportional to the effect on LDL-C elevation. Therefore, LDL-C rather than sitosterol may have been the key driver of accelerated atherosclerosis.

Taken together, accumulating evidence, including cases of infants with phytosterolemia and elevated LDL-C as well as progression/regression of xanthomas, reveal that the elevation of LDL-C may be the major cause for the development of atherosclerosis, and not elevated PS concentrations [1].

### 3.3. Phytosterol Concentrations in Tissues and Risk of Atherosclerosis

Interestingly, an increase in plasma PSs does not lead to specific enrichment of PSs in tissues, as the PS-to-cholesterol ratio is identical in plasma and in all tissues examined, including xanthomas, arterial walls, and aortic valves [22,67]. An exception is the human brain since it is not or only marginally accessible for PS [47]. Feeding mice with a PS-enriched diet over 6 weeks resulted in an increase in the concentration mainly of campesterol in the brain [68]. However, this does not correspond to the results in humans. In autopsies of patients with phytosterolemia, the brain was explicitly excluded from PS accumulation [47]. In line with these observations, there are no neurological dysfunctions reported in patients with phytosterolemia.

Plasma and tissue from elective endarterectomies of patients, who had consumed foods enriched with PSs, showed, as expected, a slightly higher PS content, while the PS-to-cholesterol ratio corresponded to that of plasma [69]. This observation holds even for individuals with homozygous phytosterolemia. While plasma PS concentrations are significantly increased in these patients, cholesterol still accounts for 80% of sterols. Hence, PSs are not over-represented in xanthomas of phytosterolemia patients; rather, they contain about 80% of sterols as cholesterol, in parallel to their plasma concentrations [22,47,60].

These findings indicate that the tissue PS concentrations merely passively follow the plasma levels, as is also the case for other lipids like fatty acids. Interestingly, despite high circulating levels, at least at a young age, sterols do not accumulate in parenchymal organs in patients with phytosterolemia but only in vessel walls, cardiac valves, tendons, and the skin [47].

The study by Horenstein et al. [70] valuated the association of being a carrier of the heterozygous G574R in ABCG8 with sub-clinical atherosclerosis as assessed by ultrasound measurement of carotid IMT. ABCG8 G574R carriers have moderately elevated plasma PS, but compared with non-carriers, decreased carotid IMT, suggesting that a moderate, life-long elevation in plasma PS is not associated with accelerated atherosclerosis and may even be protective.

In summary, there is no evidence that PSs are deposited preferentially of cholesterol in the vessel wall, nor is there evidence for a stronger atherogenicity of PSs compared to cholesterol. Epidemiologic and genetic studies in humans without phytosterolemia or heterozygous variant carriers revealed no evidence of an increased risk of atherosclerosis in individuals with moderately elevated plasma PS concentrations.

### 3.4. Phytosterolemia to Evaluate Effects of Dietary Phytosterol Intake

As outlined above, phytosterolemia has often been advocated as a model to infer the possible effects of dietary PSs. The changes in blood lipids seen in affected patients, however, mainly appear to be due to a compromised biliary secretion of PSs and cholesterol rather than an increased intestinal uptake of PS. This has been learned from studies following liver transplantation as a treatment of phytosterolemia [71]. Increased cholesterol absorption and impaired biliary cholesterol secretion in phytosterolemia may be compensated by a markedly diminished to non-measurable endogenous cholesterol biosynthesis with unchanged bile acid synthesis, which counteracts hypercholesterolemia at least in adults [45].

In individuals with phytosterolemia, plasma PS concentrations increase relatively more than plasma cholesterol concentrations. So, what can be learned from cases with phytosterolemia, except that the possible effects of dietary PSs are debatable? First, plasma PS concentrations of 10–65 mg/dL (0.3–1.2 mmol/L) have never been reached by the consumption of foods enriched with PS. Next, phytosterolemia leads to the accumulation not only of the two main PS sitosterol and campesterol but also of other xenosterols such as stigmasterol, brassicasterol, and avenasterol, which may have various effects on their own, even at low concentrations [19].

In phytosterolemia, the circulating concentration of cholesterol quantitatively preponderates and can reach considerable levels. This phenomenon is especially prominent in infant and child cases, as discussed above. In children with homozygous phytosterolemia, extremely high cholesterol levels have been observed in the range similar to those with severe homozygous FH, which can lead to fatal myocardial infarction as early as 5 years of age [10,14,29,36,72]. Therefore, phytosterolemia has also been referred to as pseudo-homozygous FH [40,57].

In a study of untreated children and adolescents with homozygous phytosterolemia aged six months to eleven years, the mean plasma sitosterol concentration was 25.5 mg/dL (0.61 mmol/L) and that of campesterol 12.8 mg/dL (0.32 mmol/L) [14]. In contrast, total cholesterol was about 490 mg/dL (12.7 mmol/L), hence a considerably stronger relative increase than that of PSs. In adults with homozygous phytosterolemia, total cholesterol concentrations were significantly lower than those of younger patients [73].

More recently, cases of phytosterolemia have increasingly been diagnosed in adults without manifest vascular disease due to other consequences of PS accumulation, mostly hematological changes. Rare exceptional cases with arthralgia, endocrine and hepatic dysfunctions have been described as well as one case of a xanthoma in the spinal canal [73,74].

### 3.5. Can Thrombocytopenia Be Used to Define a Threshold of Phytosterol Toxicity?

Hematological manifestations of phytosterolemia, such as macrothrombocytopenia with bleeding tendency, hemolytic anemia with stomatocytes and splenomegaly, have been described in children and adults because of symptoms or incidental findings [22,33,43,44,45,46,47,48,49,51]. Apparently, these symptoms are direct consequences of the alteration of the cell membrane by very high PS concentrations, as has been demonstrated experimentally [50]. In plants, PSs are the equivalent of cholesterol, from which they differ only by modifications in the aliphatic side chain. The incorporation of PS into cell membranes may lead to increased rigidity, which may explain particularly the hematological sequels of pathologically high PS concentrations, but not necessarily all their effects [13,52,72,75,76,77,78,79,80,81,82,83,84].

Macrothrombocytopenia appears to be a sensitive marker for increased PS concentrations. In 13 Chinese adult patients with phytosterolemia and a mean age of 44 years (23 to 61 years) from eight unrelated families, common leading symptoms included macrothrombocytopenia and hemolytic anemia with stomatocytes. In eight of these patients, xanthomas were found, and only two had coronary heart disease, although serum sitosterol and stigmasterol concentrations averaged 58 and 55 mg/dL, respectively. Also, five of these patients had LDL-C concentrations of 200 mg/dL and higher, i.e., in the range of heterozygous FH [36,53]. These symptoms improved following cholesterol reduction with cholestyramine and a diet with reduced PS intake.

A reduction of circulating PSs with ezetimibe can increase platelet count and decrease platelet volume. Based on clinical experience and experimental studies, macrothrombocytopenia normalizes around a PS concentration of ≤15 mg/dL, while pathological changes are observed at levels of >15 mg/dL [50,85]. The pharmacological reduction of PSs, in addition to the reduction of cholesterol, is certainly useful once plasma levels reach the toxic range >15 mg/dL [86,87]. However, ezetimibe may not fully normalize thrombocytopenia in every case. In addition, ezetimibe does not appear to be effective in infants and young children, possibly due to insufficient glucuronidation at this age [59].

The threshold of >15 mg/dL plasma concentration is so high that an intake of PSs with enriched foods is not likely to have an influence on thrombocytes or on the osmotic fragility of erythrocytes [88]. In homozygous phytosterolemia, the toxic concentration of PSs between 15 and 30 mg/dL, according to clinical criteria, can usually be undercut by appropriate nutrition and other adequate therapy, thanks to the apparent change in the response to the genetic predisposition in adulthood [86]. In heterozygous individuals, this toxic range is never reached, just as in the case of non-affected individuals, even if the consume a PS-enriched diet [20,27,28,61,62].

## 4. Phytosterols in Animal Models for Atherosclerosis

A possible relationship between circulating PS concentrations and CV risk was also investigated in various animal models [3,89]. Mice with a defect of the ABCG5/G8 transporter do not develop atherosclerosis [90]. Feeding of very high doses of PS to LDL receptor knockout mice (LDR +/−) and apolipoprotein E knockout mice (ApoE−/−) was associated with reduced development of atherosclerotic plaques [91,92,93]. The experimental PS application in this mouse model corresponded to an intake in humans of 200–250 g per day, which makes it difficult to compare with the effects possibly achievable with PS intakes of 2–3 g daily in humans. Severe toxic effects are reported only in mice with a double knockout of both the ABCG5 and the ABCG8 transporter, in which plasma PS levels rose to 250 mg/dL by feeding a PS-rich chow diet and in which PSs constituted 75% of the total sterols [83]. Toxic reactions under those artificial conditions are not surprising since, otherwise, nature would not have designed multiple mechanisms for the elimination of PSs from mammals.

Nevertheless, the effectiveness of PS consumption for reducing atherosclerosis is supported by the results of animal experiments [89], which would not occur if PSs were more atherogenic than cholesterol. Still, PSs may be as atherogenic as cholesterol on a molar basis since ezetimibe administration which lowers PSs to some extent, appears to be more effective than PS feeding in reducing the development of atherosclerotic plaques at equal total cholesterol concentrations in mice [92]. However, the concentrations of atherogenic lipoproteins have not been determined in those experiments. Furthermore, no additional effect of ezetimibe beyond that mediated via LDL-C reduction has been observed in clinical trials in humans [94,95].

There are yet other reasons that the results from animal experiments cannot be extrapolated to humans; in rodents, most xenosterols and cholesterol are transported in HDL rather than in LDL, and LDL may not be the major atherogenic particles in these animals. Further, not all aspects of murine atherosclerosis are identical to humans [96], and the regulation of lipoprotein metabolism differs vastly from that in humans in that, e.g., mice respond conversely to drugs like statins and fibrates [96,97]. Apart from that, many experiments have been carried out with excessively high PS doses.

## 5. Are plasma Phytosterol Concentrations Associated with Cardiovascular Risk?

### 5.1. Evidence from Observational Studies

Based on the suspicion of increased CV risk in individuals with phytosterolemia [47], several observational studies utilizing different cohorts assessing the association between plasma PS concentration and CV risk were carried out. The PROCAM (PROspective CArdiovascular Münster) study revealed a positive correlation between plasma sitosterol but not campesterol concentrations and CV risk [98]. In contrast, in the LASA (Longitudinal Ageing Amsterdam) study [99], the CORA (The COronary Risk factors for Atherosclerosis in women study) study [100], a Finnish study [101], and the Spanish EPIC cohort study [102], increasing plasma PS concentrations were correlated with a low CV risk. In a study with younger participants, an increasing PS-to-cholesterol ratio was associated with lower IMT of the carotid arteries as a surrogate marker of atherosclerosis [103]. The LURIC (Ludwigshafen Risk and Cardiovascular Health) study [104,105] and the Framingham Offspring Study [106] both found a weak positive correlation between plasma PS concentrations and CV risk. In contrast, in the Finland Helsinki Businessmen cohort study, higher PS concentrations in middle-aged men predicted a longer life expectancy [101]. A meta-analysis including 17 studies (sample size ranging from 40 to 2440, with 11,182 study participants in total) revealed no consistent correlation between plasma PS concentrations and CV risk [107].

**Figure 2:**
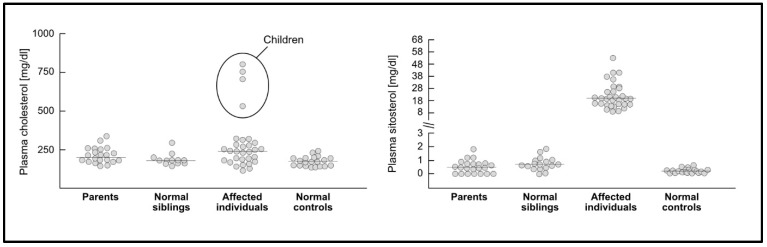
Plasma concentrations of total cholesterol and sitosterol in heterozygous (parents) and homozygous phytosterolemia cases versus unaffected siblings and unrelated controls (according to [28]).

The heterogeneity of these observational findings may be explained by differences in the prevalence of numerous factors that influence the physiological plasma concentrations of cholesterol and PS. In healthy individuals, plasma concentrations of campesterol may vary between 0.28 and 1.19 mg/dL and those of sitosterol between 0.12 and 0.66 mg/dL. Thus, the total of these two quantitatively dominating PS varies between 0.39 and 1.85 mg/dL [16]. Based on published data, individuals heterozygous for phytosterolemia reached maximum PS concentrations of up to 3.0 mg/dL, while in homozygotes or combined heterozygotes, PS concentrations were up to 65 mg/dL (Figure 2). Factors that influence the plasma concentrations of cholesterol and PS are, for example, the apolipoprotein E-phenotype and polymorphisms in the ABCG5/G8 transporters as well as in NPC1L1, but above all, different non-standardized analytical methods for the measurement of PSs in plasma or serum [16]. Such factors may impact the reported concentrations of cholesterol and PSs as well as their ratio. The differences in PS intake in omnivores, vegetarians, or vegans have only a minimal influence on the plasma PS concentrations, while cholesterol may substantially differ [108]. Individuals with diabetes mellitus and metabolic syndrome have lower plasma PS concentrations, possibly due to lower intestinal cholesterol absorption, while the effects of these conditions on CVD risk are the opposite [16]. Statins and ezetimibe lower cholesterol, while ezetimibe lowers PSs as well but does not decrease CV risk more than statins when normalized to the magnitude of LDL-C reduction [94,109].

In fact, the positive associations between PSs and CV risk, which were observed in some studies, may be because PSs are surrogate markers of the uptake of any sterols, including cholesterol, from the intestine [103,104,106]. A cross-sectional study with 270 asymptomatic individuals found a direct association of campesterol with carotid atherosclerotic plaques and an inverse association with the rates of cholesterol synthesis/absorption, suggesting that increased absorption and decreased synthesis of cholesterol, in general, are associated with carotid atherosclerosis [110].

### 5.2. Evidence from Genetic Epidemiology

Genetic variations in sterol transporters, which cause increased plasma concentrations of PSs, not only result in higher uptake and higher plasma concentrations of PSs but also in pleiotropically increased intestinal cholesterol absorption and subsequently higher plasma concentrations of LDL-C [111,112]. This may explain the correlation between increased plasma concentrations of PSs and CV risk in some Mendelian randomization studies [113]. The differences in the concentration of PS-transporting lipoproteins can have a fundamental impact on CVD patients compared to healthy controls as well. For example, higher HDL-cholesterol concentrations in healthy study participants may result in higher PS concentrations compared to patients with CVD and low HDL cholesterol [100]. So far, none of the observational studies distinguished between phytosterols in HDL and LDL particles.

Results from Mendelian randomization studies of mutations in the genes of the ABCG5/G8 transporter have been repeatedly interpreted as showing a causal relationship between increased phytosterol absorption and coronary heart disease, even though these transporters regulate the uptake of all xenosterols, i.e., dietary non-cholesterol sterols, and of dietary cholesterol [111]. In the case of such pleiotropy, the principle of Mendelian randomization only allows us to derive correlations with the gene of interest but not with one of several gene products or one of their several functions. In recognition of this limitation, the interpretation of the Mendelian randomization studies needs to be revised in that they do not prove a causal link between phytosterol concentrations and CVD [114].

Utilizing data from three studies of individuals of European origin from Iceland, Denmark, and the UK Biobank, Helgadottir et al. [115,116] investigated whether variability in cholesterol and PS absorption impacts the risk of CVD, using sequence variants of the ABCG5/8 genes. They concluded that the impact of ABCG5/8 variants on CVD cannot be fully explained by non-HDL-cholesterol and that other sterols, e.g., PSs, may contribute directly to atherogenesis. This conclusion, namely that the unexplained CVD risk is caused by absorbed PSs, has been questioned [117]. Moreover, plasma concentrations of cholestanol, a validated biomarker of intestinal cholesterol absorption, have not been reported. This would allow us to differentiate the impact of elevated PS concentrations versus increased intestinal overall sterol absorption on CVD risk.

A recent genome-wide meta-analysis of 32 PS traits reflecting cholesterol absorption, synthesis and esterification confirmed established associations between CVD and ABCG5/8 and ABO (blood group gene) next to showing an extended locus heterogeneity at ABCG5/8 with different functional mechanisms [118]. Subsequent Mendelian randomization analyses revealed a risk-increasing causal relationship between serum sitosterol concentrations and CVD, which was partly mediated by cholesterol. A direct and indirect causal effect of sitosterol on CVD risk was concluded [118].

In contrast, the results of the Copenhagen City Heart Study (CCHS) and the Copenhagen Ischemic Heart Disease Study (CIHDS) support the hypothesis of a relationship between absorbed cholesterol and CV risk [119]. It was shown that single nucleotide polymorphisms (SNPs), which increase sterol uptake and thus also intestinal cholesterol absorption, also result in reduced biliary cholesterol excretion and increased plasma LDL-C concentrations, which in turn are associated with an increased risk of coronary events and a reduced risk of gallstone formation [119]. It is noteworthy in this context that the increase in LDL-C was sufficient to explain the increased CV risk [119].

High cholesterol absorption has also been associated with increased CV risk in hemodialysis patients [120] and with the occurrence of in-stent restenosis [121].

Taken together, the findings from Mendelian randomization studies do not provide convincing evidence that the metabolic effects on CVD risk of variants in ABCG5/8 and others (ABO) are mediated by plasma PSs but rather by LDL-C or, more specifically, by an increased flux of intestinal cholesterol at the expense of hepatic de novo cholesterol biosynthesis even at a given steady state LDL-C concentration.

Essentially, three non-cholesterol surrogate markers have been suggested for the intestinal input of cholesterol into the body, namely the ratios of sitosterol, campesterol or cholestanol to cholesterol. None of them reflects cholesterol excretion into the bile. While sitosterol and campesterol are poorly absorbed plant sterols (phytosterols), cholestanol is a cholesterol degradation metabolite. While ideally, all of them should be reported, the cholestanol to cholesterol ratio is preferable since it is not affected by the supply of plant sterols with the food.

So far, none of the Mendelian randomization studies has so far reported cholestanol concentrations, which would have helped to distinguish between the risks conferred by PSs or by a high overall rate of sterol absorption. Thus, any PS-CVD association may also be explained by higher cholesterol absorption and reciprocally lower cholesterol synthesis rates which become measurable by surrogate markers such as sitosterol, campesterol and cholestanol for cholesterol absorption and plasma/serum lathosterol and desmosterol for cholesterol synthesis [122].

## 6. Do Phytosterol-Enriched Foods Increase the Risk of Atherosclerosis?

An important question is whether PSs can increase the risk of CVD when the average intake of about 300 mg per day with a habitual diet is increased to 2.5–3 g per day through PS supplements or PS-enriched foods. Several decades ago, the safety of the administration of up to 45 g/day of PSs (in non-esterified formulation) was tested over 16 weeks, and doses of up to 18 g/day were used particularly in pediatric medicine for decades to treat hypercholesterolemia [123,124,125]. However, this clinical experience can certainly not rule out an atherogenic effect of slightly increased plasma PS concentrations. In recent years, a daily PS intake of up to 9 g/day with fortified foods was tested. Daily PS doses of 2–4 g/day were administered in clinical studies for up to 85 weeks without clinically apparent adverse effects. [126,127]. Yet, the duration of these trials was certainly too short to allow conclusions about long-term atherogenic effects.

**Figure 3:**
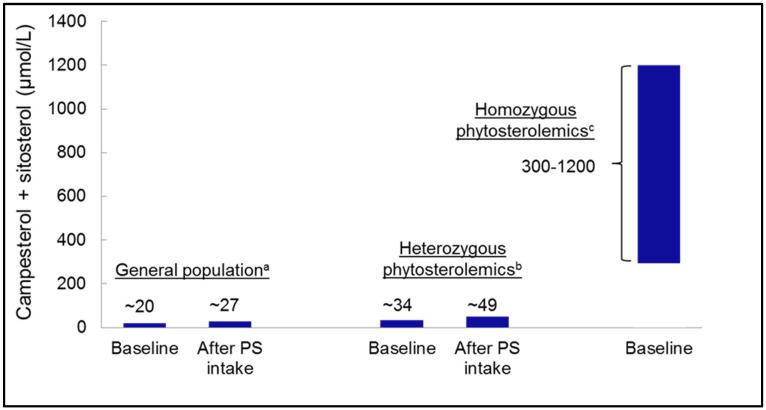
Plasma concentrations (baseline and after phytosterol (PS) intake) of campesterol and sitosterol in the general population and in individuals heterozygous for phytosterolemia versus in patients homozygous for phytosterolemia. ^a^ based on data from 41 human intervention studies [128]. ^b^ based on four studies regarding individuals with heterozygous phytosterolemia [18,61,62,129]. ^c^ based on cases with homozygous phytosterolemia [20,21].

Through the redundant defense mechanisms described above, the plasma concentrations of PSs increase only slightly following consumption of PS-containing supplements or fortified foods and never reach levels as high as seen in patients with homozygous phytosterolemia (Figure 3). A meta-analysis of 41 randomized controlled trials with 55 treatment groups in a total of 2084 participants showed that an average PS intake of 1.6 g/day in the form of fortified foods increases plasma concentrations of sitosterol and campesterol by on average 31% and 37%, respectively [128]. In absolute terms, this corresponds to an increase in plasma concentrations of sitosterol of 2.24 µmol/L (0.092 mg/dL) and of campesterol of 5.00 µmol/L (0.20 mg/dL). This compares to a reduction of total cholesterol or LDL-C of 5.9% or 8.5%, respectively, corresponding to an absolute decrease of 0.36 mmol/L (13.9 mg/dL) and 0.33 mmol/L (12.8 mg/dL), respectively. [128]. In the highest PS dose range (2.0–3.2 g/day), sitosterol and campesterol were raised by an average of 3.56 µmol/L (0.15 mg/dL) and 7.64 µmol/L (0.31 mg/dL), respectively. Thus, the proportion of PSs remained clearly below 1% of the total sterol content in the blood.

Taken together, the LDL-C lowering effect of 6–12% achieved by the consumption of PS-enriched foods reflects a decrease of plasma cholesterol that is 20–40 times higher than the parallel occurring increase in the PS concentration. Thus, PSs would need to have a 20–40 times higher atherogenicity than cholesterol to cancel the positive effect of cholesterol reduction. Yet, there is no apparent evidence for this. Based on the knowledge of the large variability of plasma PS concentrations, ranging from 0.50 to 2 mg/dL, a higher intake or higher plasma concentrations of PSs, do not necessarily indicate a higher risk for atherosclerotic CVD [130]. This is in line with the findings of a meta-analysis of observational studies, which failed to show any association between plasma PS levels concentrations and CV events [107]. Average concentrations of circulating PS for the first vs. third tertiles were 0.17 and 0.57 mg/dL (4 and 12 µmol/L) for campesterol and 0.13 and 0.38 mg/dL (3 and 10 µmol/L) for sitosterol, which is about a three-fold difference [107]. For comparison, plasma PS concentrations at baseline and after several weeks of intake of foods with added PS increased from 0.52 to 0.71 mg/dL (13–18 µmol/L) for campesterol and from 0.29 to 0.38 mg/dL (7–9 µmol/L) for sitosterol [128]. This magnitude of variation in circulating PS concentrations was covered by the meta-analysis of Genser et al. [107], which found no association.

The increases in dietary PS intake or in the plasma PS-to-cholesterol ratio, respectively, caused by vegetarian diets or by taking statins, are associated with reduced CV risk and support the concept that minor increases in plasma PS concentrations do not trigger atherosclerosis. This is in line with the experience that no increased cardiac risk is known for the heterozygosity for phytosterolemia despite doubling plasma PS concentrations [6]. A lack of effect on CV risk is further reflected in intervention studies with ezetimibe, in particular the IMPROVE-IT and the HIJ-PROPER trials [94,131]. These studies showed no further reduction of CV events that goes beyond what can be expected by the LDL-C reduction alone despite the additional PS reduction. Interestingly, statin intake has been found to be associated with increased PS concentrations in plasma and muscle tissue despite the reduction in CV risk [109,132,133].

## 7. Conclusions

The synopsis of published data shows that phytosterolemia is primarily a pediatric disease that manifests in early infancy and may extend into late adolescence. In homozygous familial phytosterolemia, atherosclerosis appears to be mainly due to extremely high plasma LDL-C concentrations during childhood, of which the CV consequences may still become manifest later in life. In some cases, hematologic changes may persist into old age. No pathological changes have been described with plasma PS concentrations less than 15 mg/dL. Therefore, phytosterolemia provides no apparent evidence that the more modest increases in plasma PS concentrations induced by the consumption of PS-enriched foods might have undesirable effects that may outweigh the 20–40-fold greater reduction of LDL-C.

Evidence from CV endpoint studies that PS consumption also lowers the risk of CVD beyond the known LDL-C lowering effect is, however, not available. Such a large-scale outcome study of foods with added PS for CVD prevention in the setting of low to intermediate risk is likely not practically feasible, given the large number of study participants (>50,000) and the long duration (˃5 years) that would be required for adequate power [3].

## Data Availability

All presented data are available in the cited literature.
